# A Modified Biogeography-Based Optimization Approach for Visual Position-Based Inverse Kinematics of Robotic Arms

**DOI:** 10.3390/biomimetics11090646

**Published:** 2026-09-09

**Authors:** Liancheng Zheng, Mohammad Soleimani Amiri, Rizauddin Ramli, Sharifah Sakinah Syed Ahmad, Xiaotian Ma

**Affiliations:** 1 School of Mechanical Engineering, Shandong Huayu University of Technology, Dezhou 253034, China; p159501@siswa.ukm.edu.my (L.Z.); maxiaotian10@gmail.com (X.M.); 2 Department of Mechanical and Manufacturing Engineering, Faculty of Engineering and Built Environment, Universiti Kebangsaan Malaysia, Bangi 43600, Selangor, Malaysia; 3 Faculty of Artificial Intelligence and Cyber Security, Universiti Teknikal Malaysia Melaka, Durian Tunggal 76100, Melaka, Malaysia; sakinah@utem.edu.my

**Keywords:** inverse kinematic, forward kinematic, biogeographical-based optimization, AprilTag

## Abstract

Precise end-effector positioning of a robotic arm is critical for accurate performance in robotics, which directly impacts the system’s performance in applications requiring high precision. This paper presents an optimized tag-based inverse kinematics approach for a 6 DoF robotic arm using a Modified Biogeography-Based Optimization (MBBO) algorithm, which is an enhanced version of the original Biogeography-Based Optimization (BBO), a population-based evolutionary algorithm inspired by the natural distribution of species across habitats. The target position is identified via AprilTag visual fiducial markers and integrated into the inverse kinematics solver. Performance was evaluated against Genetic Algorithm (GA), Particle Swarm Optimization (PSO), and BBO through 3-dimensional simulations involving ten target points. Results show that MBBO achieves reduced positioning error compared to GA, PSO, and BBO, resulting in lower end-effector position errors. The findings highlight the effectiveness of combining visual tag detection with advanced optimization for precise robotic arm control.

## 1. Introduction

Through the continual advancement of robotic technology, a variety of robot types are becoming increasingly integrated into our everyday lives, assisting humans in a wide range of applications [1,2]. Achieving precision in the end-effector positioning of a robotic arm is crucial in robotics. This can directly impact the system’s performance in applications requiring high precision, such as assembly, surgery, and manipulation because it enhances the robot’s efficiency, safety, and overall effectiveness in complex operational environments. The robotic arm is one of the most common types of robots that has been used in numerous industries like automotive, agriculture, and biomedicine. Its flexibility, strength, and accuracy make it ideal for handling complex tasks, such as avoiding joint limits and obstacles [3,4].

To replicate the intricate structure of the human arm, robotic arms are designed with manipulators made up of several segments and joints, generally divided into three main parts: the arm, wrist, and gripper [5]. These components allow robotic arms to perform delicate operations with high precision. Moreover, integrating computer vision significantly enhances their capabilities by enabling them to perceive and interpret visual information [6]. Through computer vision, visual inputs such as images or videos are transformed into meaningful data, allowing robots to identify objects, monitor motion, and respond to changes in their surroundings [7]. In robotics, computer vision is extensively utilized across various domains, including automotive manufacturing, medical technology, and automated inspection [8]. By incorporating computer vision algorithms, robotic systems can process real-time data to accurately control their movements, a capability essential for tasks involving object recognition and manipulation [9].

The utilization of fiducial marker detection systems is a precise method for acquiring the position and orientation of specific tags in relation to a camera, as documented in several studies [10,11,12]. Due to its cost-effectiveness, accessibility, and accuracy, this technique has gained prominence in drone applications. For instance, Tang et al. [13] developed an algorithm for drone applications, incorporating a range of sensors, including multiple cameras and a 2-D laser scanner, with AprilTag arrays serving as the calibration target. Zhao et al. [14] investigated how the number and spatial distribution of fiducial markers affect the accuracy of robot-guided implant placement in edentulous mandibular models. Their study demonstrated that marker quantity is a key factor for achieving high-precision robotic implant surgery, whereas marker placement strategy is less critical within the tested configurations.

Discovering the inverse kinematics solution is a crucial factor in achieving precise robot control and end-effector positioning, ultimately enhancing the accuracy of robot path following [15,16,17]. Different solutions for inverse kinematics have been explored for the robotic arm. Xu et al. [18] presented a fusion of brain–computer interface and computer vision techniques to steer a robotic arm’s end-effector to a predefined position with the assistance of a depth camera. An experiment was conducted with a 6 Degree-of-Freedom (DoF) robotic arm that received an initial desired trajectory from the user’s brain signals, combined with point clouds produced by a laser-scanning sensor.

Biogeography-Based Optimization (BBO) is an evolutionary algorithm inspired by biogeography principles, used effectively for solving complex optimization problems including inverse kinematics and trajectory planning of robotic arms. It has advantages in global search capability and convergence speed compared to classical methods like Genetic Algorithm (GA) and Particle Swarm Optimization (PSO) [19]. Serat et al. [20] explored artificial Intelligence-based computational methods, including the artificial immune system and multi-layer perceptron BBO, to calculate joint angles for a 6 DoF robotic arm. Their simulation results demonstrate that these approaches effectively handle the complexity of inverse kinematics, proving their reliability and practicality for robotic applications. Ren et al. [21] proposed a Hybrid BBO (HBBO) algorithm that combines BBO with differential evolution to efficiently solve the inverse kinematics problem of an 8 DoF redundant humanoid manipulator. By integrating a hybrid migration strategy and Gaussian mutation, the HBBO improves convergence speed, enhances population diversity, and achieves accuracy in minimizing end-effector position errors.

From a biomimetic perspective, the proposed optimization framework is motivated by the ability of natural ecosystems to achieve efficient distribution, adaptation, and survival of species under changing environmental conditions. BBO translates these ecological principles into a computational search mechanism by representing candidate solutions as habitats and modeling the migration of information among habitats through immigration and emigration processes. In the present study, this biological analogy is extended through the Modified BBO (MBBO), in which adaptive migration parameters, mutation, and elite preservation are employed to reproduce the exploration, adaptation, and diversity-maintenance characteristics observed in natural ecosystems. These biomimetic principles are particularly suitable for the inverse kinematics of robotic manipulators, where multiple feasible joint configurations must be explored while satisfying physical joint constraints and minimizing end-effector positioning error. Therefore, the proposed approach establishes a direct connection between biological adaptation mechanisms and robotic motion optimization, demonstrating how nature-inspired computational principles can be translated into an engineering solution for precise robotic positioning.

The main contribution of this paper is the development of an optimized solution for inverse kinematics by MBBO, in which the parameters of its operators are changed by increasing the iteration to provide a wider search for the global optimum during the initial iterations and narrow the search space while the algorithm reaches its final iterations. In addition, an AprilTag-based fiducial marker detection approach was employed to achieve precise end-effector positioning in a 6 DoF robotic arm.

Therefore, this study provided an effective solution for accurately determining the joint angles corresponding to the desired end-effector position. The combination of visual tag detection and advanced optimization enhanced both the accuracy and adaptability of the robotic arm’s motion control.

The novelty of this paper is the development of the MBBO algorithm for solving position-based inverse kinematics for a 6 DoF robotic arm with AprilTag to detect the end-effector position. The contributions of this paper are as follows:

- An optimal position-based inverse kinematics had been developed based on MBBO for a 6 DoF robotic arm to ensure that the end-effector reaches the target point.
- The integration of the MBBO algorithm and Tag-based visual fiducial marker systems to detect the desired target position for the end-effector precisely.
- Simulations comparing the optimal position-based inverse kinematics solved by MBBO with other conventional optimization methods such as GA, PSO, and BBO.

The rest of the paper is structured as follows: Section 2 elaborates on the optimal position-based inverse kinematics and visual fiducial marker systems. Section 3 covers the results and discussion. Section 4 concludes this paper.

## 2. Optimal Position-Based Inverse Kinematics Solution

This section presents an optimal position-based inverse kinematics solution for a 6 DoF robotic arm using MBBO. The proposed method is integrated with a visual fiducial system to track the end-effector position in real time.

### 2.1. Forward Kinematic

**Figure 1:**
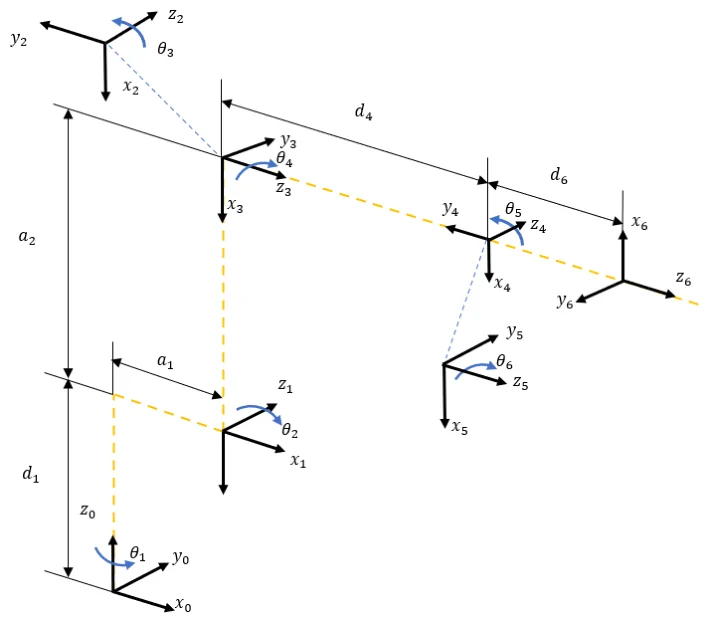
Kinematic structure of the robotic arm.

The forward kinematics of the 6 DoF robotic arm involved finding the end-effector’s position. This robotic arm was composed of a base, four links, a wrist, and a gripper, all connected in series through joints. It consisted of three rotary joints (1, 4, and 6) and three hinge joints (2, 3, and 5). Figure 1 represents the joints and kinematic structure of the robotic arm.

In this study, Denavit–Hartenberg (DH) algorithm was employed to systematically model the kinematic structure of the robotic arm. The DH algorithm provided a standardized method to define coordinate frames for each joint and link of the robotic arm, enabling precise representation of the spatial relationships between successive links. Table 1 details the specific DH parameters used the robotic arm including the link lengths, link twists, link offsets, and joint angles for each of the six joints.

In Table 1, $\alpha_{i}$, $\theta_{i}$, $d_{i}$, and $a_{i}$ denote the twist angle, joint angle, link offset, and link length, respectively. The transformation matrix of the end-effector regarding the base frame is determined by multiplying of the transformation matrix each frame to its previous frame, shown as follows:

(1) T60=T10T21T32T43T54T65

where T60∈R4×4 represents the transformation matrix of the end-effector with respect to the base frame. The *x*, *y*, and *z* positions of the end-effector in Cartesian coordinates relative to the reference frame are shown as follows:

(2) $$x=C_{1}a_{1}+d_{6}\left(C_{5}C_{1}S_{2^{\prime }3}-S_{5}\left(S_{1}S_{4}-C_{4}C_{1}C_{2^{\prime }3}\right)\right)+d_{4}C_{1}S_{2^{\prime }3}-C_{1}C_{2}a_{2}$$

(3) $$y=S_{1}a_{1}+d_{6}\left(C_{5}S_{1}S_{2^{\prime }3}-S_{5}\left(C_{4}S_{1}C_{2^{\prime }3}+C_{1}S_{4}\right)\right)+d_{4}S_{1}S_{2^{\prime }3}-C_{2}S_{1}a_{2}$$

(4) $$z=d_{1}+d_{4}C_{2^{\prime }3}+a_{2}S_{2}+d_{6}\left(C_{5}C_{2^{\prime }3}-C_{4}S_{5}S_{2^{\prime }3}\right)$$

where $C_{i}$ and $S_{i}$ denote the cosine and sine functions of the joint angle $\theta_{i}$ and $C_{i}$ and $S_{ij}$ represents cosine and sine of the joint angle $\theta_{i}+\theta_{j}$. Also, $\alpha_{i}$, $d_{i}$, and $a_{i}$ are determined based on the dimensions of the robotic arm, while $\theta_{i}$ is determined in real-time by an optimization algorithm.

In this paper, we used the ABB-IRB140 robotic arm, which is a compact and highly precise 6 DoF industrial manipulator widely utilized for assembly, handling, and research applications [22]. Based on the physical features of the ABB-IRB140 robotic arm, the DH parameters in Table 1 are defined as follows: $a_{1}=70$ mm, $a_{2}=360$ mm, $d_{1}=352$ mm, $d_{4}=380$ mm and $d_{6}=65$ mm.

### 2.2. Optimal Position-Based Inverse Kinematic

This paper investigated position-based inverse kinematics as an optimization problem, with the goal of achieving the desired end-effector position by minimizing the objective function of the ABB-IRB140 robotic arm. Optimal position-based inverse kinematics refers to solving the position-based inverse kinematics problem through an optimization-based method rather than relying only on analytical equations. This approach is particularly important for 6 DoF robotic arm, where multiple joint configurations may satisfy the same end-effector position or where the robot may approach singularities and joint limits. By formulating the problem as an optimization task, the algorithm evaluates the solutions and selects the joint configuration that provides the best performance in terms of accuracy of the end-effector position. Therefore, the use of optimal position-based inverse kinematics ensures more reliable end-effector positioning and improves the overall behavior of the robotic arm during operation [23]. The objective function is formulated as the Euclidean position error between the current and desired end-effector positions, expressed as:

(5) $$E_{p}=\sqrt{e_{x}^{2}+e_{y}^{2}+e_{z}^{2}}$$

where $e_{x}$, $e_{y}$, and $e_{z}$ are the tracking error of the end-effector position represented as follows:

(6) $$e_{x}=x-x_{d}$$

(7) $$e_{y}=y-y_{d}$$

(8) $$e_{z}=z-z_{d}$$

where $x_{d}$, $y_{d}$, and $z_{d}$ represent the target end-effector positions in relation to the reference frame. Meanwhile, *x*, *y*, and *z* represent the end-effector current position, determined using forward kinematics.

The position-based inverse kinematic formulation was selected because the objective of the present study is point-to-point positioning of the robotic-arm end-effector, where the desired target is specified by its Cartesian position obtained from the AprilTag detection system. Therefore, the optimization objective focuses on minimizing the translational position error in the *x*, *y*, and *z* directions. Although a 6 DoF manipulator generally provides both position and orientation control, orientation constraints are not required for the point-to-point positioning task considered in this study. The resulting six joint angles provide a valid robot configuration for reaching the desired Cartesian target position while satisfying the joint-angle limits.

Various metaheuristic algorithms such as PSO and GA can be used for the optimization problem [24,25]. In this study, the MBBO was selected to solve the inverse kinematics, which is a nature-inspired optimization algorithm that draws inspiration from the study of biogeography based on the distribution of species and ecosystems across geographical space and time [26].

MBBO is an improved version of the conventional BBO, which is a population-based evolutionary algorithm inspired by the natural distribution of species across habitats. It enhances the original approach through mutation as an extended migration mechanism and by introducing dynamic operator adaptation, which helps to prevent premature convergence and avoid local optima.

Unlike the conventional BBO, the proposed MBBO incorporates several modifications to improve the search capability of the optimization process. First, a mutation mechanism is introduced as an extended migration operation to increase population diversity. This mechanism promotes exploration of new regions of the search space and reduces the likelihood of the population becoming trapped in local optima. Second, adaptive parameters $\alpha_{1}$ and $\alpha_{2}$ are employed to dynamically adjust the optimization operators during the search process. This dynamic adaptation provides a balance between exploration and exploitation, allowing the algorithm to explore promising regions during the early stages while increasingly exploiting high-quality solutions as the optimization progresses. Third, an elite preservation strategy is incorporated to retain high-quality solutions between iterations. By protecting the best solutions from being lost during population updates, elite preservation strengthens exploitation and supports stable convergence toward an optimal or near-optimal solution. Therefore, the combination of mutation, adaptive operator adjustment, and elite preservation enables MBBO to improve population diversity, balance exploration and exploitation, avoid premature convergence, and achieve more stable convergence than conventional BBO.

**Figure 2:**
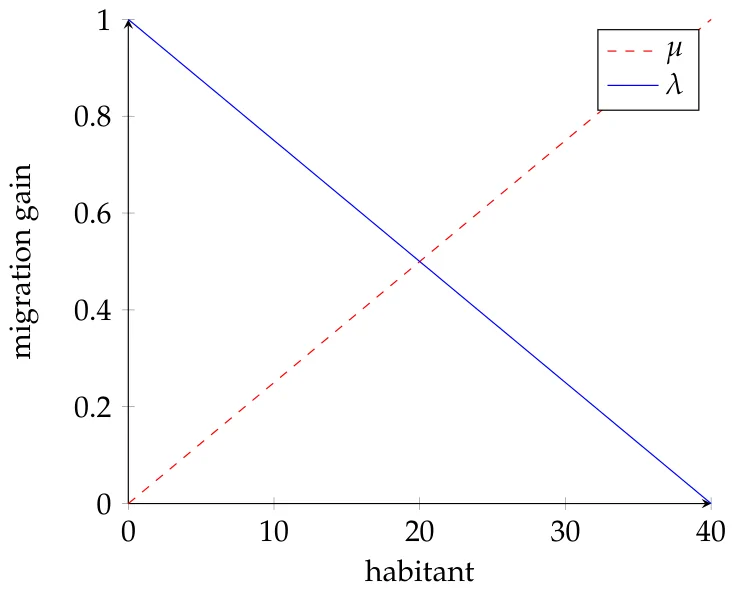
Migration rates in the population.

The initial value of the MBBO was chosen randomly, and the number of populations or ’habitats’ was usually determined by the designer. Although various methods, such as the Taguchi experimental design method, have been used to determine the population size in evolutionary algorithms, this paper took a trial-and-error approach and set the population size at 40. The population in the proposed MBBO is given as follows:

(9) $$x_{i,j}=\left[\begin{array}{cccccc} \theta_{11} & \theta_{12} & \theta_{13} & \theta_{14} & \theta_{15} & \theta_{16}\\ \theta_{21} & \theta_{22} & \theta_{23} & \theta_{24} & \theta_{25} & \theta_{26}\\ \theta_{31} & \theta_{32} & \theta_{33} & \theta_{34} & \theta_{35} & \theta_{36}\\ \vdots & \vdots & \vdots & \vdots & \vdots & \vdots \\ \theta_{40,1} & \theta_{40,2} & \theta_{40,3} & \theta_{40,4} & \theta_{40,5} & \theta_{40,6} \end{array}\right]$$

where i=1,2,…,40 is the number of habitats, and j=1,2,…,6 is suitability index variable, which are design variables for MBBO, i.e., joint angles. Afterward, the habitats were evaluated by calculating the objective function and then sorted in ascending order. 20% of the habitats, referred to as the elites, were kept unchanged, while the remaining habitats changed migration and mutation processes to maintain diversity. This strategy ultimately improved the MBBO’s ability to discover global optima and prevented it from becoming trapped in local optima. The migration involved emigration and immigration rates, as illustrated in Figure 2.

In Figure 2, λ and μ represent immigration and emigration rates assigned to each individual in habitats. The initial habitat displays the lowest emigration rate and the highest immigration rate, creating a paradox where emigration is at its highest, while immigration is at its lowest. The migration operator is implemented by generating a random value and comparing it with the λ of all habitats. If the generated random number, *r* is smaller than λ, the migration operator is employed to establish new habitats for the upcoming iteration. Otherwise, the inhabitants of the previous generation remain unaltered. The migration operator to update the habitat for the next iteration is inspired by blend crossover, represented as follows:

(10) $$if\;r_{1}<\lambda_{i}:\;x_{i,j}^{t+1}=\omega \left(\left(x_{k,j}^{t}+\alpha_{1}\right)-\left(x_{i,j}^{t}\right)\alpha_{2}\right),\;j=1,2,\ldots ,6$$

where $r_{1}$ is a uniformly distributed random number in the range (0,1) and *t* is the iteration number. ω, $\alpha_{1}$, and $\alpha_{2}$ are the parameters of the migration operator. $x_{k,j}$ is a selected habitat for creating new offspring habitat for the new iteration. The selection of *k* is determined using a roulette wheel scheme based on migration probabilities, as given by

(11) $$p_{i}=\frac{\mu_{i}}{\sum_{n=1}^{40}\mu_{n}},\;i=1,2,\ldots ,40,$$

where $p_{i}$ denotes the migration probability of the (i)-th habitat. The cumulative migration probability is given as follows:

(12) $$C_{i}=\sum_{n=1}^{i}p_{n},\;i=1,2,\ldots ,40.$$

A random value is chosen from the roulette wheel and compared with each element in $C_{i}$. The first element of $C_{i}$ that surpasses the random value is denoted as *k*, as defined in Equation (10). The roulette wheel algorithm increases the likelihood of selecting habitats with a higher emigration rate.

The parameters of the migration operator, including ω, $\alpha_{1}$, and $\alpha_{2}$, are not constant throughout all iterations. This adaptive behavior initially broadens the exploration of the search space during the early iterations and progressively narrows it down toward the global optimum as the number of iterations increases, as follows:

(13) $$\omega =\omega_{min}+\left(w_{int}-w_{min}\right)e^{-3a}$$

(14) $$\alpha_{1}=2.3\cdot b+2.6$$

(15) $$\alpha_{2}=2.3\cdot a+0.1$$

where $\omega_{int}$ is the initial parameters for ω, alternatively. *b* and *a* are dynamic parameters that vary within the range [0,1], with *b* decreasing and *a* increasing over iterations, as described below:

(16) $$a=\frac{t}{t_{\max}},\;\mathrm{for}\;t=1,2,\ldots ,t_{\max}$$

(17) b=1−a

where $t_{\max}$ represents the maximum iteration value. The $\omega_{int}$ and $\omega_{min}$ are set at 1.2 and 0.3. This provides gradual refinement around the optimum for Equation (13).

This design enables the MBBO to explore a broad search space during the early iterations and gradually narrow its focus toward the global optimum in later iterations. Such an adaptive strategy enhances the algorithm’s performance by increasing early-stage iterations to prevent local optima convergence and achieve more precise solutions.

**Figure 3:**
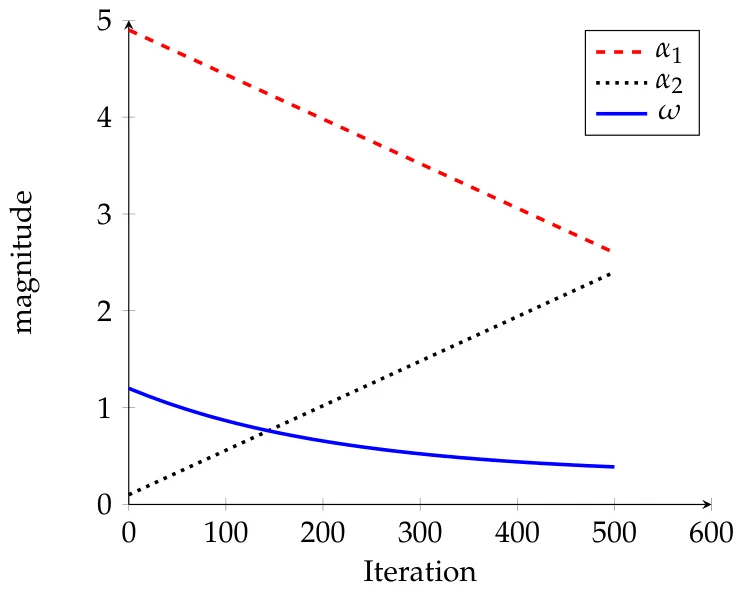
Changes in ω, $\alpha_{1}$, and $\alpha_{2}$ across all iterations.

The sum of $\alpha_{1}$ and $\alpha_{2}$ remains constant at 5 throughout all iterations, with $\alpha_{1}$ decreasing and $\alpha_{2}$ increasing. Figure 3 illustrates the variations in migration parameters as the number of iterations changes. The maximum number of iterations is set to 500.

In addition to the migration process, the mutation operator plays a crucial role in generating new solutions within the MBBO framework. The mutation probability, $m_{p}$, is a predefined positive constant less than one, given as follows:

(18) $$m_{p}\in \left[0,1\right]$$

A randomly generated value, *r*, which is applied in each iteration, is compared with $m_{p}$, and when it is less than *r*, mutation is applied to the selected habitat. The mutation is mathematically defined as follows:

(19) $$if\;r_{2}<m_{p}:\;x_{k,j}^{t+1}=x_{i,j}^{t}+\sigma_{j},\;\mathrm{for}\;j=1,2,\ldots ,6$$

where $r_{2}$ is a uniformly distributed random number in the range (0,1). $\sigma_{j}$ is independently generated randomly for each joint variable according to a uniform distribution. Therefore, the mutation operator can either increase or decrease the corresponding joint angle, thereby maintaining population diversity and eliminating directional bias during the search process. $x_{k,j}$ is the selected habitat by the roulette wheel scheme to apply the mutation.

Since the optimization variables correspond to the robot joint angles, all candidate solutions are constrained within the physical joint limits of the ABB-IRB140 robotic arm. After each migration and mutation operation, a boundary-check mechanism is applied to ensure that every joint variable satisfies $\left(\theta_{j}^{\min}\leq \theta_{j}\leq \theta_{j}^{\max}\right)$. Any variable exceeding its upper or lower bound is clipped to the corresponding limit. This guarantees that all habitats represent feasible robot configurations throughout the optimization process. The lower and upper bounds of each joint are determined according to the mechanical motion limits of the ABB-IRB 140 robotic manipulator, as listed in Table 2. During the optimization process, any candidate solution is limited to the corresponding boundary to ensure physically feasible joint configurations.

After the migration, mutation, and angle boundary-check operations, all offspring habitats are evaluated using the objective function and sorted in ascending order. Let

(20) $$X^{off}=\left\{x_{1}^{off},x_{2}^{off},\ldots ,x_{i}^{off}\right\},$$

denote the offspring population after sorting, where $x_{1}^{off}$ represents the best habitat. Likewise, the elite habitats preserved from the previous generation are defined as

(21) $$e^{t}=\left\{x_{1}^{t},x_{2}^{t},\ldots ,x_{i_{e}}^{t}\right\},$$

where $i_{e}$ denotes the number of elite habitats. The new population is then obtained by replacing the worst $i_{e}$ offspring habitats with the elite habitats from the previous generation according to

(22) $$x_{k}^{t+1}=\left\{\begin{array}{cc} x_{r}^{off}, & r=1,2,\ldots ,i-i_{e},\\ x_{\;r-(i-i_{e})}^{t}, & r=i-i_{e}+1,\ldots ,i. \end{array}\right.$$

Algorithm 1 shows the pseudo code of MBBO.
**Algorithm 1** Pseudo code of MBBO  1:Start;  2:Generate the initial population of 40 habitats;  3:Evaluate all habitats;  4:Sort the habitats in ascending order;  5:Set the iteration counter t=1;  6:Set initial parameters for *r*, μ, λ, and $m_{p}$;  7:**while** $t<t_{\max}$**do**  8:    Store the top 20% habitats as elite solutions;  9:    **for** i=1 to 40 **do**10:        Generate a random number r∈(0,1);11:        **if** $r_{1}<\lambda_{i}$ **then**12:           Select habitat *k* using roulette wheel selection;13:           Compute ω, $\alpha_{1}$, and $\alpha_{2}$;14:           Update habitat by migration operator:15:              $x_{i,j}^{t+1}=\omega \left(\left(x_{k,j}^{t}+\alpha_{1}\right)-\left(x_{i,j}^{t}\right)\alpha_{2}\right)$;16:        **end if**17:        **if** $r_{2}<m_{p}$ **then**18:           Mutate habitat according to:19:              $x_{k,j}^{t+1}=x_{i,j}^{t}+\sigma_{j}$;20:        **end if**21:        Apply angle boundaries:22:           $x_{i,j}=max\left(\theta_{j}^{\min},min\left(x_{i},\theta_{j}^{\max}\right)\right)$;23:    **end for**24:    Evaluate all habitats;25:    Sort the habitats in ascending order;26:    Replace the worst habitats with the elite habitats;27:    Update the current population with the modified habitats;28:    t=t+1;29:**end while**30:Select the top-ranked habitat as the best solution;31:End;

The maximum iteration $t_{max}$ is 500. In this algorithm, the term $\left(x_{k,j}^{t}+\alpha_{1}\right)$ is the scalar combination inside the habitat of the migration operator. The significance of MBBO compared to the conventional BBO is that its operation parameters vary by iteration, as mentioned in Figure 3 and Equations (13)–(15). This provides a wider search for the global optima during the initial iterations and narrows the search space while the algorithm reaches its final iterations. The advantages of MBBO are the faster response and lower chances of being trapped in local optima. This also provides lower positioning error for the final result.

### 2.3. Visual Fiducial Marker System

In this study, visual target detection was achieved by equipping the robotic arm with a camera rigidly attached to its end-effector. The camera continuously captured images of the workspace, where AprilTag fiducial markers are placed at target locations. The AprilTag detection algorithm processes these images in real-time, estimating the unique ID and precise 3-Dimensional (3D) position and orientation of each tag relative to the camera frame using established perspective-n-point techniques [13]. Knowing the fixed transformation between the camera and the end-effector, the detected tag pose was transformed from the camera frame to the robot’s coordinate system, thereby defining the desired end-effector position for the manipulator. This spatial information served as the input for the inverse kinematics solver, which computes the corresponding set of joint angles needed to drive the end-effector to the detected tag pose. By integrating visual perception with kinematic modeling, the robotic arm can autonomously and accurately adjust its posture to reach visually identified targets, enabling robust and adaptable manipulation within simulated environments.

Numerous visual fiducial marker systems, including ArUco, ARTag, and ARToolKit, are widely used in various applications. Among them, AprilTag has gained popularity in robotics applications due to its robustness and flexibility. These systems are employed to detect and distinguish 2D markers from one another [27].

In this paper, the AprilTag marker of the 36H11 family is utilized. The raw camera image is processed to detect the position and orientation of the marker relative to the camera frame [28]. This process can be divided into two main components: the tag detector and the code system. The AprilTag system extracts unique ID information embedded in the marker and estimates the position of the marker relative to the camera [23].

**Figure 4:**
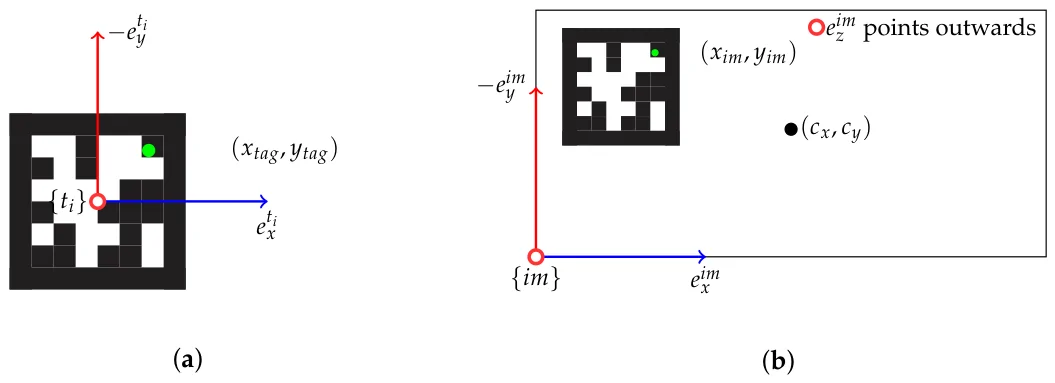
Coordination of a point in both tag marker and image frames: AprilTag Marker ID:1 (**a**) Image frame coordination (**b**) Image frame coordination.

Let $H_{i}\in R^{3\times 3}$ be the homography matrix corresponding to tag i∈D, where *i* is the marker ID from the set of detected tags *D*. The homography $H_{i}$ projects a point $\left(x_{\mathrm{tag}},y_{\mathrm{tag}},0\right)$ from the coordinate frame of tag *i*, denoted as $\left\{t_{i}\right\}$, to the pixel coordinate $\left(x_{\mathrm{im}},y_{\mathrm{im}}\right)$ in the image frame {im} [29]. Figure 4 illustrates the coordinate representation of a point in both the AprilTag marker and the image frame.

The homography matrix $H_{i}$ is represented as follows:

(23) $$H_{i}=PE$$

where,

(24) $$\left[\begin{array}{ccc} h_{11} & h_{12} & h_{13}\\ h_{21} & h_{22} & h_{23}\\ h_{31} & h_{32} & h_{33} \end{array}\right]=\left[\begin{array}{cccc} f_{x} & 0 & c_{x} & 0\\ 0 & f_{y} & c_{y} & 0\\ 0 & 0 & 1 & 0 \end{array}\right]\left[\begin{array}{ccc} R_{11} & R_{12} & T_{x}\\ R_{21} & R_{22} & T_{y}\\ R_{31} & R_{32} & T_{z}\\ 0 & 0 & 1 \end{array}\right]$$

where $P\in R^{3\times 4}$ is the perspective projection matrix, which is obtained through the camera calibration process. $f_{x}$ and $f_{y}$ are the focal lengths, while $c_{x}$ and $c_{y}$ are the principal points, typically located near the center of the image frame {im}. $E\in R^{4\times 3}$ represents the extrinsic matrix, where $R_{ij}$ denotes the elements related to rotation, and $T_{x}$, $T_{y}$, and $T_{z}$ are the translation components. Generally, the extrinsic matrix E is a 4×4 matrix, but the third column is omitted for computational efficiency [30]. The relationship between the AprilTag marker frame and the image frame is given as follows:

(25) $$\left[\begin{array}{c} x_{im}^{\prime }\\ y_{im}^{\prime }\\ z_{im}^{\prime } \end{array}\right]=H_{i}\left[\begin{array}{c} \frac{x_{tag}}{s_{tag,t}}\\ \frac{y_{tag}}{s_{tag,t}}\\ 1 \end{array}\right]$$

The vector obtained in Equation (25) is represented in homogeneous image coordinates. The corresponding Cartesian image coordinates are obtained through homogeneous normalization by dividing the first two components by the third component, given as follows:

(26) $$\left[\begin{array}{c} x_{im}\\ y_{im} \end{array}\right]=\left[\begin{array}{c} \frac{x_{im}^{\prime }}{z_{im}^{\prime }}\\ \frac{y_{im}^{\prime }}{z_{im}^{\prime }} \end{array}\right]$$

Here, $s_{\mathrm{tag},i}$ represents the half-size length of tag *i*. Since the AprilTag frame $\left\{t_{i}\right\}$ and the camera body frame {B} are defined with different origins and axis orientations, the estimated tag pose must be transformed from the tag coordinate frame to the camera body frame through the corresponding rigid-body transformation [31].

## 3. Results and Discussion

**Figure 5:**
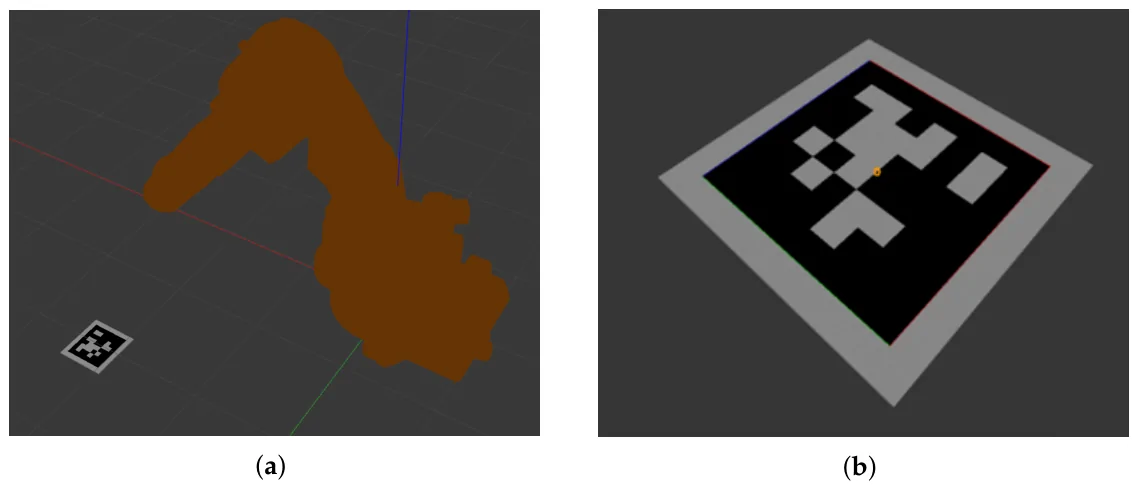
Simulated robotic arm in a virtual environment. (**a**) Robotic arm with AprilTag. (**b**) Camera View of AprilTag.

To evaluate the performance of the optimal inverse kinematics using MBBO, a 3D model of the 6 DoF robotic arm is simulated within a virtual environment. The simulated robotic arm and camera view of the AprilTag are captured from a camera that is rigidly attached to the robotic arm end-effector is shown in Figure 5.

In this simulation, the robotic arm’s end-effector was commanded to move from its target position to a target location identified by an AprilTag marker. The tag’s position was accurately detected through the AprilTag vision system and established as the desired target position for the task. This target position is then incorporated into the objective function of the optimal inverse kinematics problem, which is solved using the MBBO algorithm. The inverse kinematics solution yielded six joint angular positions, corresponding to the 6 DoF of the robotic manipulator. These angular positions served as control inputs applied to the simulated robotic arm within the virtual environment.

Since the robotic arm and the attached camera are simulated in a virtual environment, the camera parameters and camera-to-robot extrinsic transformation are defined directly within the simulation environment. Therefore, physical camera calibration and extrinsic calibration procedures are not performed in this study.

The camera is rigidly attached to the end-effector link; therefore, its position coincides with the end-effector position in the simulation. The AprilTag target position is initially obtained with respect to the camera coordinate frame and is transformed into the robotic-arm reference coordinate frame before being provided to the inverse kinematics solver. Since the camera is rigidly mounted on the end effector, the relative camera-to-end-effector transformation is fixed and predefined in the simulation.

Regarding AprilTag localization error, the present study evaluates the system in a virtual environment; therefore, real-world sources of localization error, such as image noise, illumination variations, lens distortion, camera resolution, and imperfect tag detection, were not experimentally evaluated. In the simulation, the detected AprilTag position is used as the target position for the inverse kinematics solver.

**Figure 6:**
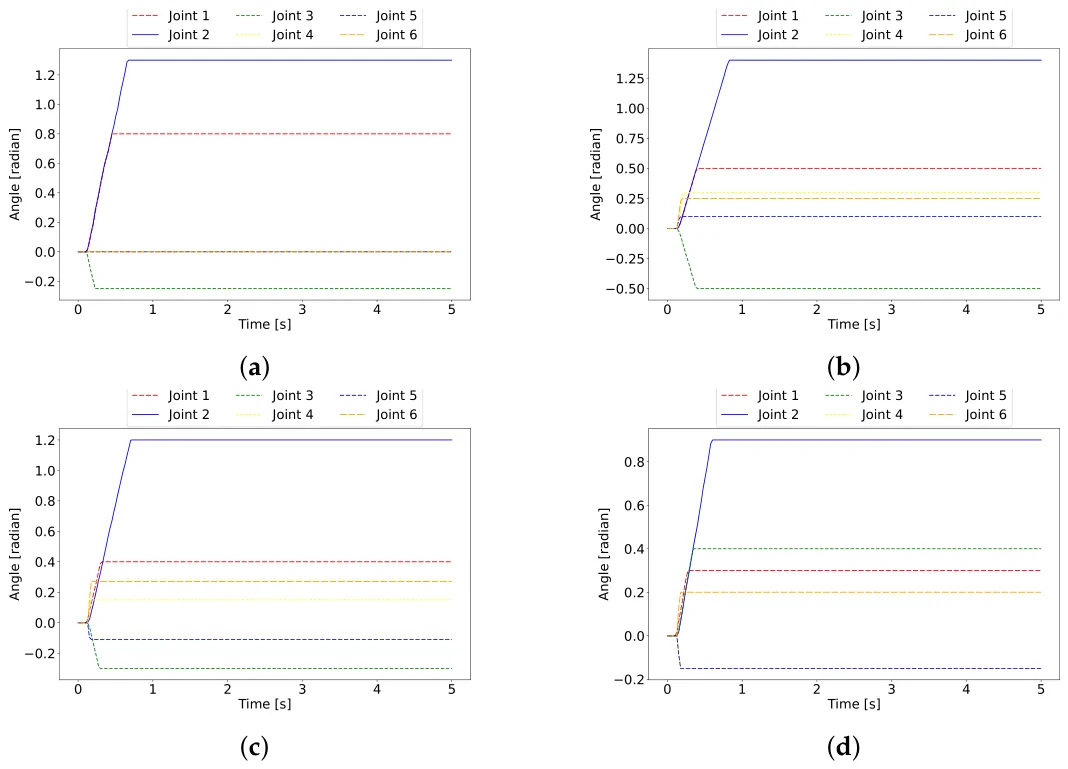
Time-domain joint responses generated by applying the optimal inverse kinematics solution obtained using the proposed MBBO algorithm for four different target points in virtual environment. (**a**) Point A, (**b**) Point B, (**c**) Point C, (**d**) point D.

The proposed MBBO algorithm computes the optimal joint angles corresponding to the target position detected by the AprilTag system. These optimized joint angles are subsequently transmitted as reference commands to the ABB-IRB140 robotic model in the virtual environment. The robot then executes the corresponding motion while the joint positions are recorded over time. Figure 6 illustrates the time-domain joint responses of the ABB-IRB140 robotic arm during the execution of the optimized inverse kinematics solution for four different target positions detected by the AprilTag system in virtual environment.

From Figure 6 it is concluded that the optimal inverse kinematics by MBBO and integrated with AprilTag performed effectively for the robotic arm inverse kinematics solution. It is found that joints 1, 2, 3, and 5 exhibit significant motion, with joint 5 reaching approximately 1.3 radians and remaining constant thereafter, indicating a stable target position has been achieved. Joint 1 stabilizes near 0.8 radians, while joint 3 moves slightly into the negative range before settling, reflecting precise adjustments for accurate end-effector positioning. Joints 4 and 6, in contrast, remain near zero throughout the motion, suggesting minimal or no movement required for this particular task. Figure 6 demonstrated the effectiveness of the MBBO algorithm in generating feasible and stable joint angle solutions that enable the robotic arm to reach the target position identified by the AprilTag with precision and without abrupt transitions or oscillations.

In this study, 10 different target points for the end-effector are applied to the simulated robotic arm model and the optimal inverse kinematic, which is solved by GA, PSO, and MBBO for better comparison. To ensure a fair comparison among the optimization algorithms, identical population sizes and maximum iteration numbers were employed for all methods. The hyperparameter settings used in this study are summarized in Table 3.

Table 4 compares the objective function for MBBO, GA, PSO and BBO.

The optimal inverse kinematics solution was evaluated using GA, PSO, BBO, and MBBO across ten target points for the robotic arm end-effector. Since metaheuristic optimization algorithms exhibit stochastic behavior, each algorithm was independently executed 30 times for every target point. The results presented in Table 4 are reported as the mean and standard deviation of the Euclidean position error.

The MBBO algorithm achieved the lowest average position error among all compared optimization methods, demonstrating improved optimization accuracy and solution stability. Compared with GA, PSO, and conventional BBO, the MBBO reduced the average position error by approximately 67.3%, 76.1%, and 32%, respectively. Statistical analysis was further performed using the Wilcoxon signed-rank test to assess the significance of the differences between MBBO and the compared optimization algorithms across the ten target points. The results showed that MBBO achieved significantly the lowest position errors and *p* = 0.00195 for GA, PSO and BBO based-on Wilcoxon signed-rank test. These results indicate that the lower position errors achieved by MBBO were statistically significant across the evaluated target points. Therefore, the statistical analysis further supports the superior positioning accuracy of the proposed MBBO compared with the conventional optimization algorithms.

The integration of AprilTag detection to identify the target position of the robotic arm’s end-effector significantly enhances the accuracy of the inverse kinematics, solved by MBBO. This improved target localization directly translates to more precise determination of joint angles, enabling the robotic arm to achieve highly accurate and reliable positioning of each joint. Consequently, the fusion of AprilTag-based visual feedback with inverse kinematics not only strengthened the overall control performance but also facilitated precise and adaptive manipulation tasks within the simulated environment. In addition, the performance of the MBBO compared to the other two conventional optimization problems showed that the MBBO provided the efficient solution for the inverse kinematics problem.

With the employment of AprilTag and the use of MBBO in determining the robotic arm’s end-effector position demonstrates its superior accuracy and efficiency compared to GA, PSO and BBO methods. This resulted in more precise joint angle solutions and improved overall control performance, confirming MBBO as a more effective approach for inverse kinematics in robotic manipulation tasks.

To further evaluate the optimization algorithms under challenging target conditions in Table 4, points K and L, which are near the workspace boundaries of the ABB IRB140, were introduced. Point K represents a near-vertical workspace-boundary condition, while Point L represents a highly extended configuration near the radial workspace boundary. Point M was considered as a challenging singular configuration corresponding to a wrist-singular condition with $\theta_{5}=0^{\circ }$.

**Figure 7:**
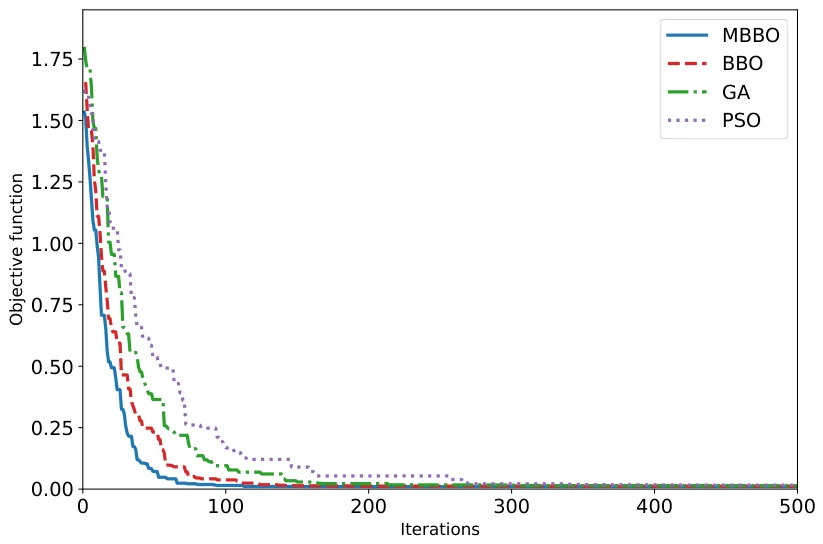
Comparison of convergence behavior of GA, PSO, BBO and MBBO algorithms.

Figure 7 illustrates a comparison of the convergence behavior of GA, PSO, BBO, and MBBO algorithms.

MBBO demonstrates a faster reduction in the objective function value and reaches a lower final objective value compared with the other algorithms. This indicates that MBBO converges more rapidly toward high-quality solutions and provides more consistent optimization performance.

The proposed MBBO algorithm successfully solves the inverse kinematics problem using the target position obtained from the AprilTag detection system. Compared with GA, PSO, and the conventional BBO algorithm, MBBO achieves lower position errors and more accurate end-effector positioning across the tested target points.

## 4. Conclusions

In this paper, an optimal inverse kinematics solution for a 6 DoF robotic arm was presented. The position of the end-effector in the Cartesian system was developed by forward kinematics. The MBBO solved the inverse kinematics problem, and its operators determined dynamic adaptation across iterations to avoid trapping in local optima and achieve a more accurate solution.

The performance of the inverse kinematics by MBBO integrated with AprilTag was examined in a virtual environment to execute point-to-point motion to a specific target point for the robot end-effector. The presented method provided an accurate and reliable inverse kinematics solution that was integrated with a visual fiducial marker system. The comparison of the MBBO as the suggested optimization algorithm for inverse kinematics solution with GA, PSO, and BBO demonstrated superior positioning accuracy for solving this problem.

The limitation of this work is by not executing the MBBO to the actual robotic arm. In future work, the path planning algorithm can be added to this algorithm to avoid the end-effector collision with the possible obstacles.

## Figures and Tables

**Table 1:** DH parameters of the 6 DoF robotic arm.

Joints	$\theta_{i}$	$d_{i}$	$a_{i}$	$\alpha_{i}$
1	$\theta_{1}$	$d_{1}$	$a_{1}$	$-\frac{\pi }{2}$
2	$\frac{\pi }{2}+\theta_{2}$	0	$a_{2}$	0
3	$\theta_{3}$	0	0	$-\frac{\pi }{2}$
4	$\theta_{4}$	$d_{4}$	0	$\frac{\pi }{2}$
5	$\theta_{5}$	0	0	$-\frac{\pi }{2}$
6	$\theta_{6}$	$d_{6}$	0	0

**Table 2:** Joint angle limits of the ABB-IRB 140 robotic manipulator.

Joint	Lower Bound (°)	Upper Bound (°)
$\theta_{1}$	−180	180
$\theta_{2}$	−90	110
$\theta_{3}$	−230	50
$\theta_{4}$	−200	200
$\theta_{5}$	−115	115
$\theta_{6}$	−400	400

**Table 3:** Hyperparameter settings for optimization algorithms.

Algorithm	Hyperparameters
GA	Population size = 40,
	Maximum iterations = 500,
	Crossover rate = 0.8,
	Mutation probability = 0.1
PSO	Swarm size = 40,
	Maximum iterations = 500,
	Inertia weight w=0.7,
	Cognitive coefficient $c_{1}=2$,
	Social coefficient $c_{2}=2$
BBO	Population size = 40,
	Maximum iterations = 500,
	Mutation probability = 0.1
MBBO	Population size = 40,
	Maximum iterations = 500,
	Elite preservation = 20%,
	Mutation probability $m_{p}=0.1$,
	Initial ω=1.2,
	Adaptive $\alpha_{1}$ and $\alpha_{2}$

**Table 4:** Comparison of position error for GA, PSO, BBO, and MBBO over 30 independent runs.

Target Point (m)	GA	PSO	BBO	MBBO
A = [0.45, 0.10, 0.50]	0.032±0.008	0.088±0.021	0.021±0.006	0.017±0.004
B = [0.30, −0.20, 0.60]	0.036±0.009	0.110±0.028	0.024±0.005	0.019±0.005
C = [0.20, 0.35, 0.55]	0.058±0.014	0.031±0.010	0.023±0.007	0.015±0.004
D = [0.40, −0.30, 0.70]	0.042±0.011	0.058±0.017	0.028±0.008	0.022±0.006
E = [0.35, 0.40, 0.45]	0.086±0.019	0.066±0.016	0.031±0.009	0.018±0.005
F = [0.25, −0.45, 0.40]	0.062±0.015	0.090±0.023	0.020±0.006	0.014±0.004
G = [0.50, 0.15, 0.35]	0.035±0.010	0.031±0.009	0.019±0.005	0.013±0.003
H = [0.40, −0.35, 0.30]	0.045±0.012	0.033±0.011	0.018±0.005	0.012±0.003
I = [0.38, −0.42, 0.32]	0.044±0.011	0.120±0.030	0.022±0.006	0.016±0.004
J = [0.30, 0.45, 0.28]	0.048±0.013	0.046±0.012	0.021±0.007	0.018±0.005
K = [0.07, 0.00, 1.092]	0.071±0.017	0.098±0.025	0.034±0.010	0.021±0.006
L = [0.765, 0.00, 0.099]	0.064±0.015	0.082±0.021	0.029±0.008	0.019±0.005
M = [−0.290, 0.000, 0.609]	0.053±0.026	0.075±0.015	0.038±0.016	0.017±0.004
Average Error (m)	** 0.052±0.021 **	** 0.071±0.034 **	** 0.025±0.010 **	** 0.017±0.005 **

## Data Availability

The original contributions presented in this study are included in the article. Further inquiries can be directed to the corresponding authors.
